# Reproductive tract microbiota of women in childbearing age shifts upon gynecological infections and menstrual cycle

**DOI:** 10.1186/s12866-021-02300-4

**Published:** 2021-09-21

**Authors:** Lijuan Cheng, Yan Gao, Qing Xia, Hui Wang, Xiuzhen Xie, Yurong Liu, Heying Shang, Yutao Diao

**Affiliations:** 1 Department of Obstetrics and Gynecology, The First Affiliated Hospital of Shandong First Medical University & Shandong Provincial Qianfoshan Hospital, Key Laboratory of Laparoscopic Technology, the First Affiliated Hospital of Shandong First Medical University, Jinan, China; 2 The Second Affiliated Hospital, Shandong University of Traditional Chinese Medicine, Jinan, 250001 China; 3 Zhangdian District Center for Disease Control and Prevention, Zibo, 255000 China; 4 Qilu Hospital of Shandong University, Jinan, 250012 China; 5 Obstetrics and Gynecology Department, The 5th People’s Hospital of Jinan, Jinan, 250022 China

**Keywords:** Microbiome, Microbiota, *Lactobacillus*, Alpha diversity, beta diversity, Relative anundance

## Abstract

**Background:**

This study was undertaken to discover whether the vaginal microbe of women at childbearing age is different among groups defined by urogenital tract infections, childbearing history and menstrual cycle, respectively.

**Results:**

This was a multiple case-control study of women at childbearing age who were assigned to case or control groups according to their states of urogenital tract infections. The participants were also grouped by childbearing history and menstrual cycle. Vaginal swabs were collected and stored at − 70 °C until assayed. The V3-V4 region of 16S rRNA gene was amplified using PCR and sequenced on the Illumina MiSeq platform. We tested the hypothesis of whether the relative abundance of microbial species in vaginal microbiota was varied with urogenital tract infections, childbearing history and menstrual cycle. The vaginal microbial richness (Alpha diversity measured by PD_whole tree) was decreased in normal women (without reproductive tract infections) than in those with bacterial vaginosis (BV), and decreased in pregnant women than in other groups of non-pregnancy. Similarly, women from groups of normal and in pregnancy had lower beta diversity on measure of unweighted_unifrac distance in comparison to those of infected and non-pregnant. The top 10 genus relative abundance, especially *Lactobacillus*, which was the most dominant genus with the relative abundance of 71.55% among all samples, did not differ significantly between groups of childbearing history and menstrual cycle analyzed by ANOVA and nonparametric kruskal_wallis. *Lactobacillus iners* and *Lactobacillus helveticus* have the most abundance, totally account for 97.92% relative abundance of genus *Lactobacillus*. We also found that a higher *L.helveticus*/*L.iners* ratio is more likely to present in normal women than in the infected and in pregnant than in non-pregnant, although these comparisons lack statistical significance.

**Conclusions:**

The relative abundance of dominant bacterial taxa in vaginal microbial communities of women at childbearing age were not different among groups of childbearing history and menstrual cycle. Women from groups of in pregnancy and without reproductive tract infections had lower alpha and beta diversity. The composition of the main *lactobacillus* species may shift upon phases of a menstrual cycle and the status of reproductive tract infections.

**Supplementary Information:**

The online version contains supplementary material available at 10.1186/s12866-021-02300-4.

## Background

Woman genital tract can harbor substantial amount of body microbes [1]. The normal structure of vaginal microbiome plays a pivotal role in maintaining the healthy vaginal microenvironment, especially for those in childbearing age. Any deviations of this eubiosis caused by direct behavioral factors, for instance, poor sanitation or unclean sexual intercourse, can lead to disorders such as bacterial vaginosis (BV), mycotic vaginitis (MV) and pelvic inflammation, which significantly impact the health of women, their fetuses and new born infants [2–4]. Furthermore, the role of dysbiosis in causing gynecological cancers has been appreciated only recently [5]. On the other hand, the composition of genital tract microbiota differs depending on factors that have no direct relation to infection, such as race, nationality and country [6–8]. In addition, a number of reports dealt with the impact of a natural menstrual cycle on bacterial growth, colonization, and community structure, but the participants were not from Chinese population [9, 10]. Other reports were about the comparison of vaginal microbial composition between non-pregnant and pregnant women by prospective case-control studies, but their results did not reflect on the real menstrual cycle [11, 12]. We thus postulate that gynecological infections as well as the normal physiological cycle may affect the structural profile of vaginal microbiota of women in childbearing age from Chinese Han nationality. So we sought to examine this question by characterizing the differences of microbiota among groups defined by urogenital tract infections, childbearing history and menstrual cycle.

## Results

The grouping and characteristics of the 111 study subjects were shown in Additional file 1: Table S1 and Fig. S1. Significant differences in age_at_first_marriage, days of menstrual_cycle, and pH_of_vaginal_discharge were not found between groups divided by childbearing history, menstrual cycle, gynecological infections, respectively. The average age of women without childbearing was significantly lower than those with childbearing and those in pregnancy.

**Fig. 1:**
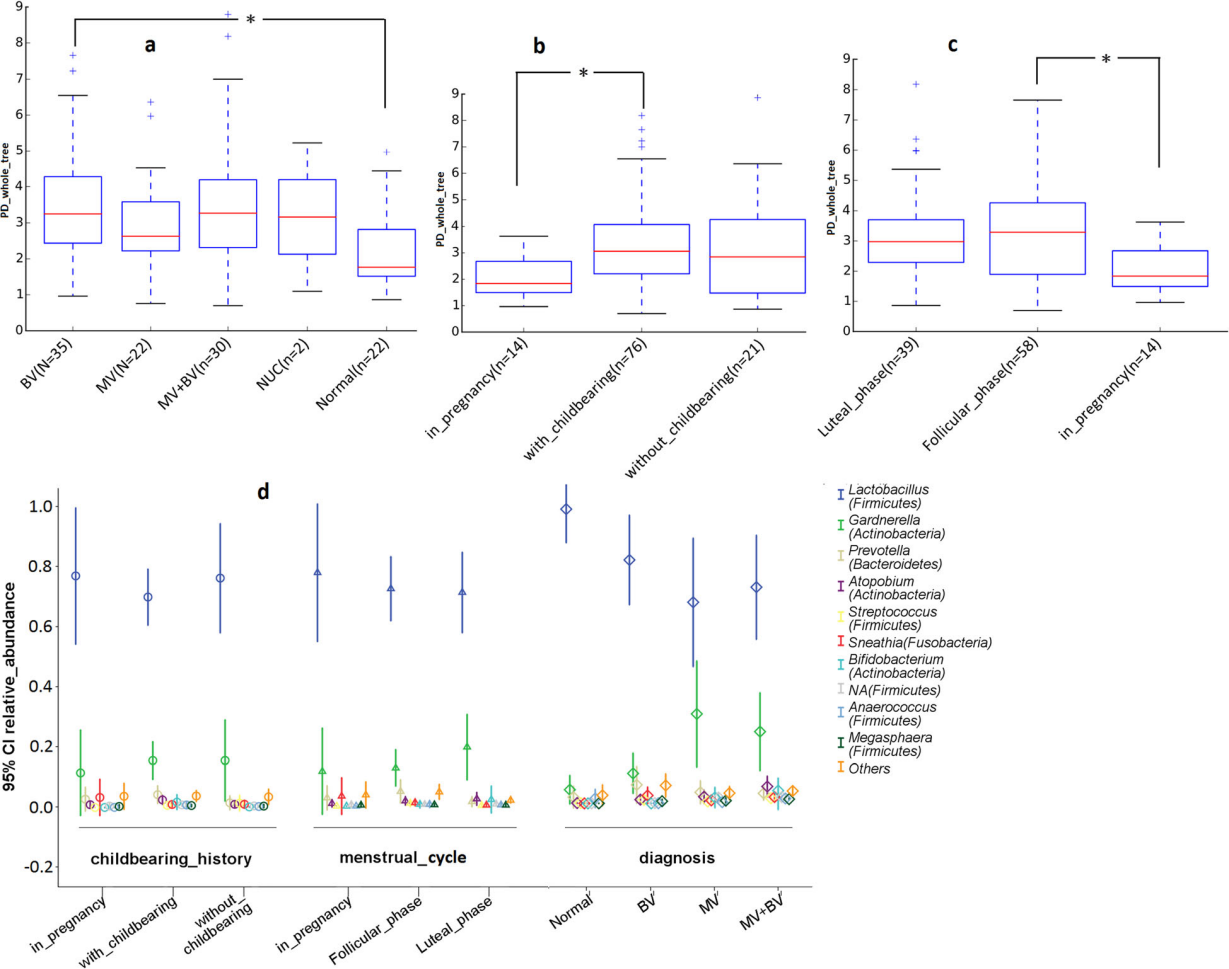
**a** Alpha diversity by PD_whole_tree was lower in Normal women than in the other groups of gynecological infections and was significantly lower in normal women than in BV positive women(*P*<0.05). **b** Women in pregnancy (from ≥40 days gestation to parturition) had significant lower PD_whole_tree than those with childbearing. **c** Women in pregnancy had significant lower PD_whole_tree than those in follicular phase. **d** Means and 95% intervals of 10 genus-level relative abundances that had no significant difference among groups by childbearing history and menstrual cycle according to both ANOVA and nonparametric kruskal_wallis with Bonferroni correction. But the relative abundances of *Gardnerella*, *Streptococcus*, and an order-level relative abundance of *Lactobacillales* (NA (Firmcutes)) were significantly different among the 5 groups of gynecological infections. In **a**, **b** and **c**, the boxes are interquartile range (IQR); median values are the bands within the boxes; the line terminals outside the boxes are upper and lower endpoint of the data; crosses are outliers. In **d**, the lines are 95% intervals of genus-level relative abundance; mean values are icons in the middle of the 95% interval lines

**Fig. 2:**
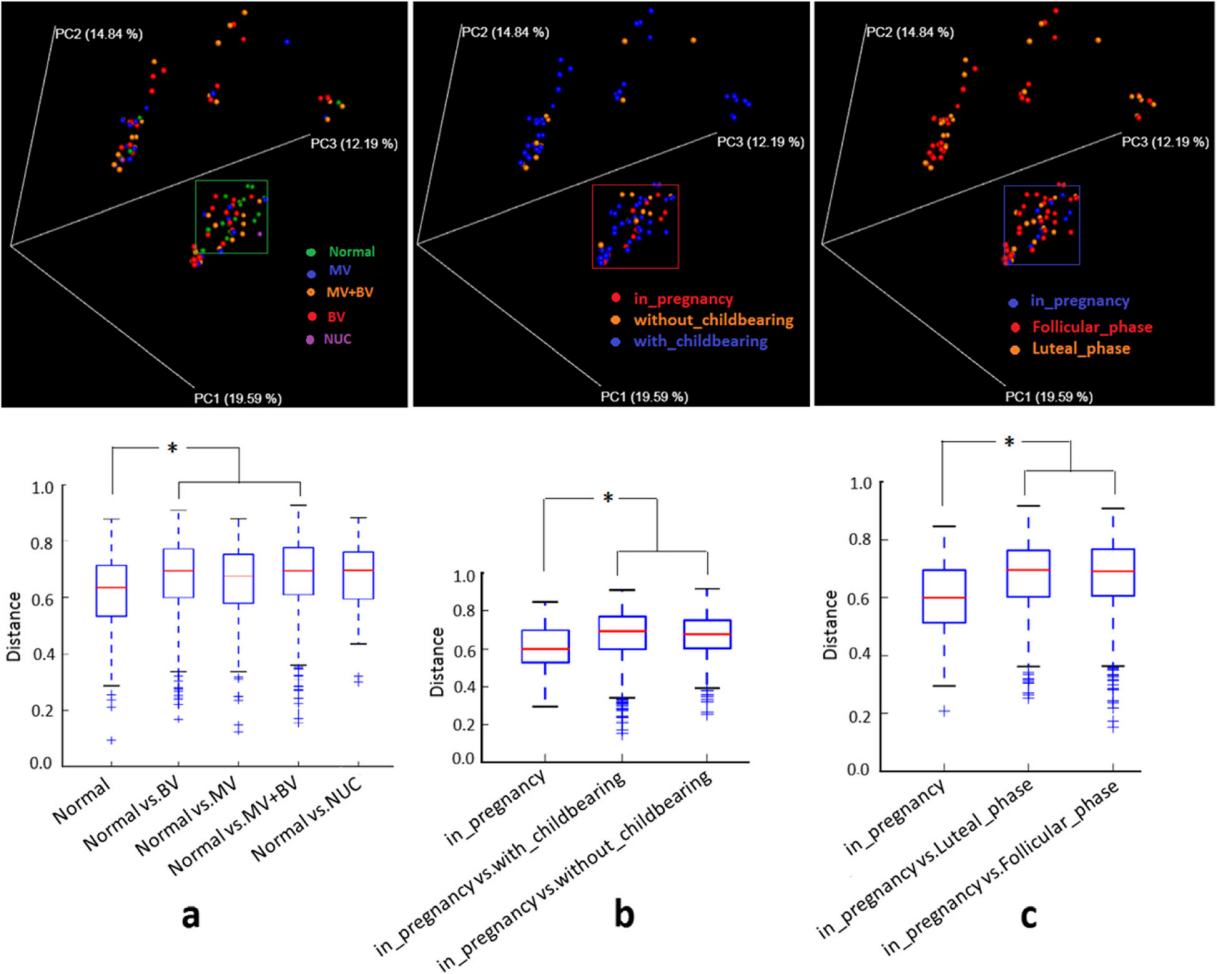
The permutation of the 111 samples in a 3-dimensional space constructed by principal coordinates analysis (PCoA) based on Unweighted UniFrac distance. **a** upper: Samples in Normal group were clustered together (green dots in the rectangle) and had significant shorter genetic distance apart from samples in the other groups of gynecological infections. **a** lower: Unweighted UniFrac distance (beta diversity) of within-group (Normal) was significantly shorter versus that of between-groups (Normal vs the other gynecological infections). **b** upper: samples from in pregnancy group were clustered together (red dots in the rectangle) apart from samples in groups of with and without childbearing. **b** lower: Unweighted UniFrac distance of within-group (in pregnancy) was significantly shorter versus that of between-groups (in pregnancy vs with or without childbearing). **c** upper: samples from in pregnancy group were clustered together (blue dots in the rectangle) apart from samples in groups of luteal and follicular phase. **c** lower: Unweighted UniFrac distance of within-group (in pregnancy) was significantly shorter versus that of between-groups (in pregnancy vs in luteal or follicular phase). In **a**, **b** and **c** lower, the boxes are interquartile range (IQR) of Unweighted UniFrac distance; median values of distance are the bands within the boxes; the line terminals outside the boxes are upper and lower endpoint of the data; crosses are outliers. The tests of significance were performed using a two-sided Student’s two-sample t-test. The nonparametric *P*-values were calculated using 999 Monte Carlo permutations

The alpha diversity on measure of PD_whole tree other than chao1, observed_outs and Shanonn’s index was lower in group of pregnant women compared to those with_childbearing (*P* = 0.021) and those in follicular phase (*P* = 0.048). Of the 5 groups defined by gynecological diagnosis, normal women had lower alpha diversity in contrast to the other groups, but only the comparison to BV group was statistically significant (*P* = 0.04) (Fig. 1a-c, Additional file 1: Table S2). As for the measurement of beta diversity, normal women were clustered together based on the fact that their genital tract microbial taxa having closer genetic affinity (having shorter unweighted_unifrac distance) apart from those that affected BV, mycotic vaginitis (MV) and MV + BV (Bonferroni-corrected parametric *P*<0.001, Bonferroni-corrected nonparametric *P* = 0.136) (Fig. 2a, Additional file 1: Table S3). With respect to childbearing history and menstrual cycle, women in pregnancy had obvious similarity of microbial population compared to those with or without childbearing (Bonferroni-corrected parametric *P* = 0.001, Bonferroni-corrected nonparametric *P* = 0.028) (Fig. 2b, Additional file 1: Table S3). Similarly, taxa in genital tract of pregnant women were significantly alienated from those that were in luteal_phase or follicular_phase(Bonferroni-corrected parametric *P*<0.001, Bonferroni-corrected nonparametric *P* = 0.028) (Fig. 2c, Additional file 1: Table S3).

**Fig. 3:**
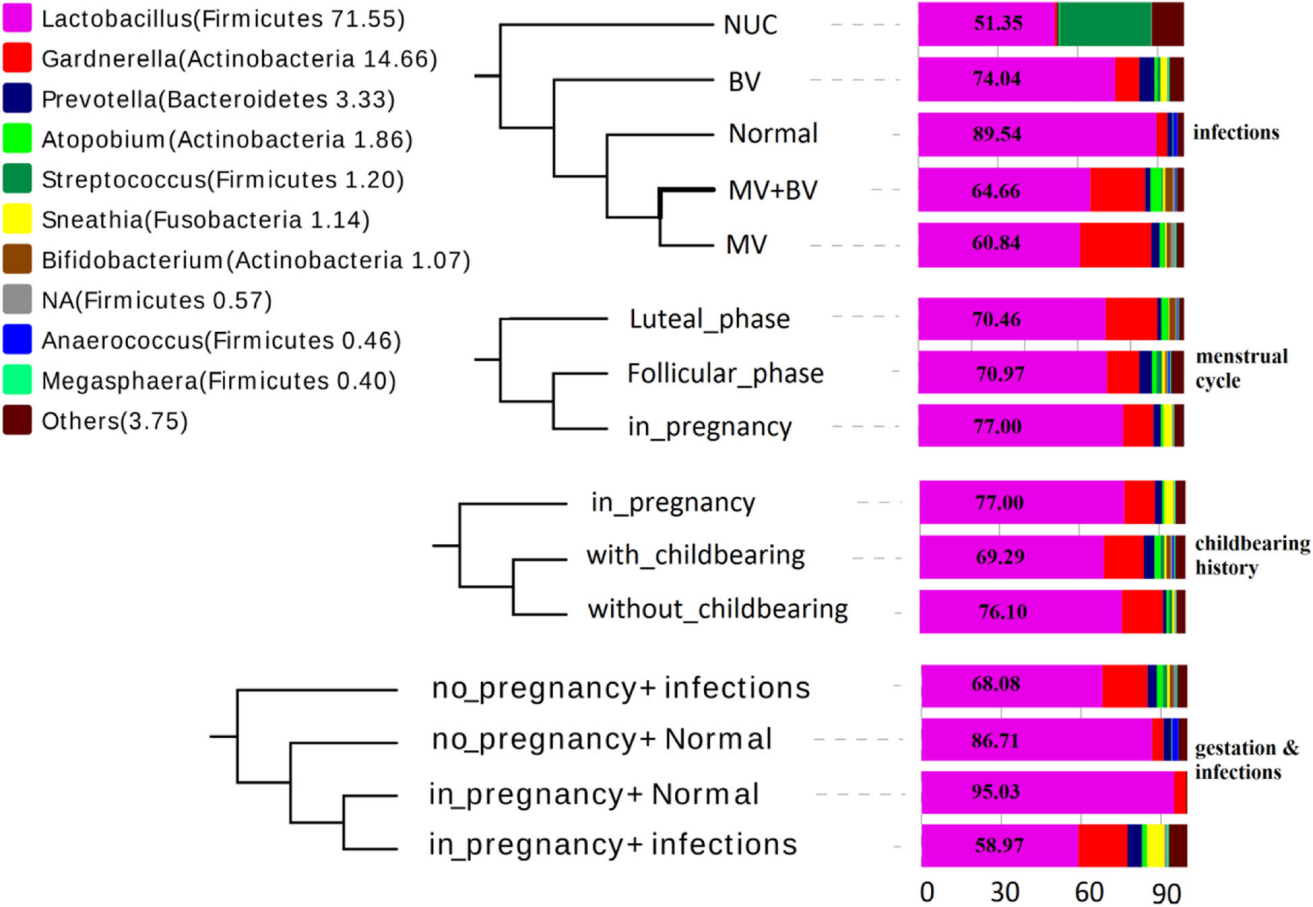
The compositional profiles of the top 10 genera and dendrogram of the 4 sample grouping schemes (participants were grouped based on infections, menstrual cycle, childbearing history and infections & with or without gestation, respectively). The left side showed the genera and phylum (in parenthesis) that the genus were affiliated to with the average relative abundance (%) of each genus in the total 111 women shown in parenthesis. The dendrogram in the middle was based on hierarchical clustering using complete linkage of Unweighted UniFrac distance of the OUT table. Each horizontal bar in the right represents the compositional profile averaged within each sample group wherein the number in each bar indicates the percentage of genus *lactobacillus*. Only the top 10 abundant genera were shown and the other genera after the top 10 ones were artificially merged into one genus named ‘Others’

The relative abundances of the top 10 genus did not differ significantly among women groups compared by ANOVA and nonparametric kruskal_wallis, with one exception that groups defined by gynecological infections had different relative abundance of 3 out of the 10 genera tested by ANOVA (Fig. 1d, Fig. 3, Additional file 1: Table S4). The detail of these differences were intensively analyzed by Bonferroni multiple comparisons. That is, MV group had higher relative abundance of genus *Gardnerella* compared to Normal group (*P* = 0.041) and had higher relative abundance of genus NA (affiliated to orders *Lactobacillales*) compared to Normal group (*P* = 0.042) and BV group (*P* = 0.025) (Fig. 1d, Additional file 1: Table S5). Women affected nongonococcal urethritis/cervicitis (NUC) had higher relative abundance of *Streptococcus* in contrast to the others (*P* < 0.001) (Additional file 1: Table S5). The graph of NUC group was not included in Fig. 1d due to its 95% interval line disproportionately expanded caused by the low sample size (only 2 women). *Lactobacillus* is the most dominant over others for sustaining normal vaginal microenvironment [13, 14]. As shown in Fig. 1d and Fig. 3, *Lactobacillus* was the most dominant genus in all the sample groups with the mean relative abundance of *Lactobacillus* being the highest in Normal group over other infection groups. But there were no significant relative abundance discrepancies between groups of gynecological infection in genus *Lactobacillus*, nor were in *Atopobium* and *Prevotella*, even though which had been classified as bacterial vaginosis associated bacteria (BVAB) or as markers for the other forms of urogenital tract infections (data not shown).

**Fig. 4:**
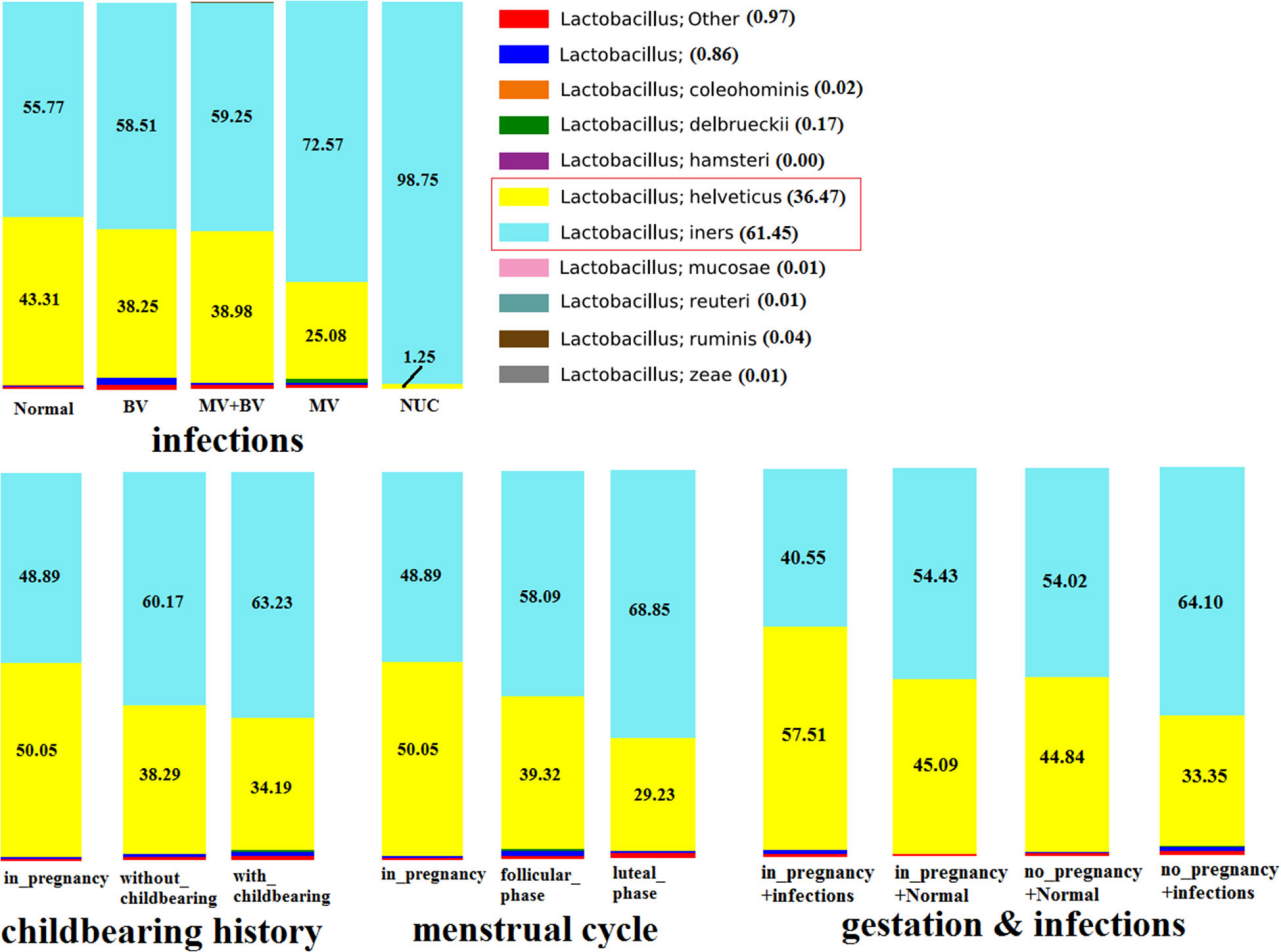
The compositional profiles of *lactobacillus* genus (including 10 known *lactobacillus* species, the unknown ones were all ascribed to a species named *lactobacillus Other*) in 4 grouping schemes (participants were grouped based on infections, menstrual cycle, childbearing history and infections & with or without gestation, respectively). The average relative abundances (%) in the total 111 women were shown in parenthesis after each species. Each bar represents the compositional profile of lactobacillus species averaged within each group with the average relative abundances (%) of the 2 most abundant species (*lactobacillus iners* and *lactobacillus helveticus*) were shown

In addition to the common sense that *Lactobacillus* is the dominant population in Normal (without infectious diseases) group of women [13, 14], we found women in gestation had a slight higher *Lactobacillus* relative abundance in comparison to nonpregnant women. Furthermore, women in gestation and without genital tract infections (in_pregnancy+Normal) harbored the highest proportion of genus *Lactobacillus* over the other groups (Fig. 3). In order to scrutinize the exquisite constituent structure of genus *Lactobacillus*, we explored the compositional profile of all the 11 *Lactobacillus* species wherein *Lactobacillus iners* and *Lactobacillus helveticus* have the most abundance, totally account for 97.92% relative abundance of genus *Lactobacillus*. Pregnant and normal women seem to have a higher proportion of *Lactobacillus helveticus*. We thus proposed that a higher *L*.*helveticus*/*L*.*iners* ratio is more likely to present in normal women than in the infected women and in pregnant ones than in nonpregnant ones, although these comparisons lack statistical significance (at 0.05 significant level) (Fig. 4).

## Discussion

In this study, **w**e established that the structural pattern of vaginal microbiota characterized by high relative abundance of *Atopobium* as well as the presence of *Prevotella*, *Sneathia*, *Gardnerella*, *Ruminococcaceae*, *Parvimonas*, *Mobiluncus* and other taxa that were previously considered to be bacterial vaginosis associated bacteria (BVAB) was rarely observed in either pregnant or other groups of women. Nevertheless, we found that *Gardnerella* and *Streptococcus* were significant indicator genera for mycotic vaginitis (MV) and nongonococcal urethritis/cervicitis (NUC), respectively. Although there were no significant differences of relative abundance of the top 10 bacterial genera, which totally account for 96.25% microbe amount of the whole microbial community, between pregnant and non-pregnant women, we observed the decreased microbial diversity measured by PD_whole tree in pregnant than in non-pregnant women and in normal than in infected ones. This results are consistent with a line of studies [15, 16] and only contrary to the result recently reported by Yulian Chen,et al. [17] in that they found both pregnancy and HPV infection could increase vaginal bacterial microbial richness and diversity. Although we considered the reason is they measured the microbial diversity by Chao and Shannon index other than PD_whole tree that focus on the phylogenic affinity between each microbe, there must be other underlying factors that obscure the real relationship between taxonomic diversity and pregnancy. Anyway, a plausible explanation is the estrogen and progesterone levels increase as the pregnancy progresses, reaching their peaks at the 3rd trimester. Additionally, the immune system dynamically strengthens during pregnancy. The elevated level of gonadal hormones along with a strengthened immune system might result in stable and less diverse vaginal microbiota during the middle and later phases of pregnancy [18]. Still, some intensive studies involving metagenome or 16S RNA gene sequencing are essential to identify the differences of microbial functional genes among the corresponding groups defined by urogenital infections or normal physiological cycle [19–21]. The results of these studies would be helpful to reasonably explain the presence of a more stable and less diverse vaginal microbiota during pregnancy.

*Lactobacillus* was undoubtedly the most dominant genus with a relative abundance of 71.55% in the 111 women in this study. Oddly, we did not find at species level the presence of *L. vaginalis*, *L. crispatus*, *L.gasseri* and *L. jensenii* as reported in previous studies from Western countries and non Asians [22–24]. Indeed, the dominant members of *Lactobacillus* species have been limited and elusive based on the context of studies conducted in different locations and different populations. In most cases, the vaginal microbiota through a menstrual cycle demonstrated that *L.crispatus*, *L.iners*, and *L.jensenii* were the dominant members [11]. *Lactobacillus gasseri* was considered prominent in other studies [22, 23], but it was not, along with *L.crispatus*, *L.vaginalis* and *L.jensenii*, a dominant organism in this study rather than *L.iners* and *L. helveticus*. Because the participants of the above mentioned studies were all from Caucasian women, intensive studies based on Asian or Chinese women are necessary to compare the conclusion of this study with that of Western studies. There has been recent species-specific attention to *L.iners* which is commonly found in the vagina [22] and has been associated with both BV and healthy states [25–28]. In addition, *L.iners* is often the first *Lactobacillus* species to recover after treatment for BV [28, 29]. But the role of *L.iners* in the dynamic balance of the family members of vaginal microbiota is still controversial. Some reports suggested that some strains of *L.iners* are highly stable over time while others are associated with a rapidly changing vaginal microbiota toward BV [23, 30]. Jakobsson et al. [31] reported that *L iners* is a dominant part of the vaginal flora when the flora is in a transitional stage from normal to abnormal while others observed that *L.crispatus* produce more H_2_O_2_ as a bacteriocide as well as other protective agents compared with other *Lactobacilli* species [14], so that it is considered the leading *Lactobacilli* strain in maintaining the health of vaginal microecosystem, even though there were other argues that lactic acid rather than H_2_O_2_ plays an important role in the antimicrobial properties of protective vaginal Lactobacillus spp. [32]. Unfortunately, we did not found *L.crispatus* in the present as well as in our previous study samples [33]. According to our findings, *L.iners* and *L.helveticus* totally accounted for 97.92% relative abundance of genus *Lactobacillus* with *L.helveticus* was discovered for the first time in this study as a leading member in genus *Lactobacillus*. Moreover, we found an increased proportion of *L.helveticus* over *L.iners* in normal and pregnant women than in other groups of women, which indicated it may be a marker of healthy microbial status of woman reproductive tract. We also conjectured that women with *L.iners*-dominant and *L.crispatus*-absent intravaginal microbial flora, who were preliminarily presumed to be normal by phenotypic methods, were actually at risk of BV or other urogenital tract infections and must be monitored carefully. Finished and ongoing works are needed to evaluate the genomic heterogeneity of *L.iners* and if different strains are associated with health and abnormal outcomes [34].

In brief, we engaged on exploring the compositional differences of vaginal microbes between groups of women at reproductive age defined by urogenital tract infections, childbearing history and menstrual cycle, respectively. The vast volume work of processing and analyzing sequencing data was performed on QIIME 2 and QIIME 1.9.1 platform embedded in Linux operation system while some outcomes resulted from QIIME was analyzed by SPSS 22.0. These methods may effective to identify significant differences among groups of women defined by urogenital tract infections, childbearing history and menstrual cycle. Weaknesses include the modest sample size, especially for NUC sample group that had only 2 women, which contributed to the larger variance in the comparison of outcomes between groups using parametric methods such as ANOVA. This study only allows us to detect the main effect size of different grouping schemes without the control of confounding factors such as age, occupation, and other behavioral factors.

## Conclusions

Women without reproductive tract infections or in pregnancy had decreased vaginal microbial richness (alpha diversity) and beta diversity. We found no bacterial vaginosis associated bacteria (BVAB) except for *Gardnerella*. The compositional ratio of the main *Lactobacillus* species may shift depending on the normal physiological cycle and reproductive tract infections and the species level profile of *Lactobacillus* were also differed from other studies. Maybe this discrepancy was due to the different source of participants on whom there must be different administration of antibiotic formulae, for instance, antimicrobials abuse is popular in present in China.

## Methods

### Study subjects

111women at reproductive age (32.61 ± 7.75 years old) were recruited at Qilu Hospital of Shandong University and the 5th People’s Hospital of Jinan city, China, and were grouped by reproductive tract infections (gynecological diagnosis), childbearing history and the phase of menstrual cycle on the day the specimen swabs were collected (Additional file 1: Figure S1).

Generally, a duration from the first day of menstruation to the day before the next menstruation is called a menstrual cycle [35]. According to the average 28 days of each menstrual cycle, 111 women of childbearing age in this study were divided into 3 stages in proportion to the average menstrual cycle:

1. Follicular_phase is equivalent to the interval from the 5th to 14th day of a menstrual cycle.
2. Luteal_phase is the period from the beginning of ovulation to the next menstruation, i.e. the 15th to 28th day of a menstrual cycle.
3. In_pregnancy was defined as from 40 days pregnancy to parturition. Because women in menstrual period (equivalent to the period from the 1st to 4th day of a menstrual cycle) could not visit the hospital, there were no women of menstrual period recruited to this study and the gynecological sampling was not available for those just in menstrual period.

The grouping of childbearing history was performed with respect to the results of questionnaire and clinical examination. Nugent score and Amsel’s criteria in combination with clinical symptoms were used to identify normal women from those with different reproductive tract infections [36]. Individuals with antibiotic usage within the last 2 weeks or those with examination and treatment of urogenital tract within the last 3 days were excluded. The study protocol was approved by the study review board and ethic committee of Institute of Basic Medicine, Shandong First Medical University & Shandong Academy of Medical Sciences. All the subjects signed informed consent and filled out questionnaires.

### Biospecimen collection

We got two swabs from each woman with one dry swab thoroughly wiped the lateral and posterior fornix of the vaginal wall to collect the complete population of microbiome, while the other physiological saline soaked swab were used to extract vaginal discharge for measurement of pH and direct detection of mycotic infection under optical microscopy.

### 16S rRNA gene sequence analysis

The DNA was extracted from the dry swab of each sample as described previously [37, 38]. The V3-V4 regions of the 16S rRNA gene were amplified using PCR and sequenced on the Illumina MiSeq platform with the Illumina RTA software for image recognition and basecalling, and Illumina bcl2fastq 2.17 software for demultiplexing at Suzhou GENEWIZ® Biotechnology Co., Ltd., China (https://www.genewiz.com/en/Public/Services/Next-Generation-Sequencing).

A total of 7,041,153 high-quality(Q30(%): 71.78–95.03) sequences was obtained with a median read count per sample of 59,560 (range: 40,173-174,385) (Additional file 1: Table S6). These single-end reads were processed for quality filtering, denoising, and chimera removal to form Amplicon Sequence Variants (ASVs, also known as Operational Taxonomy Units (OTUs)) using the DADA2 plugin [39] in QIIME2(Quantitative Insights into Microbial Ecology, version: 2019.10 (1572640561)) [40]. OTUs with only one read were excluded.

Alpha diversity was estimated by the measure of observed_otus, Shannon’s Index (using information of OTU frequency) [41], chao1 and PD_whole_tree(using information of phylogenetic relationship of different OTUs) [42] using a sampling depth range of 10–25,000 seqences/sample to generate 10 rarefied OTU tables and compute alpha diversity metrics for each rarefied OTU table. Beta diversity was measured by unweighted UniFrac, weighted UniFrac and bray_curtis distance based on the 25,000 seqences/sample rarefied OTU table [43]. Relative abundances of taxa summarized at the L2(Phylum) to L6(genus) levels were also respectively calculated from the 25,000 seqences/sample rarefied OTU table. These analyses were conducted using the work flow *core_diversity_analyses.py* in QIIME (version 1.9.1, [44]) and Green genes data based classifier (version gg-13-8-99-nb) (http://data.qiime2.org/2019.10/common/gg-13-8-99-nb-classifier.qza) as the taxonomic reference.

### Statistical analysis

The tests of alpha diversity (PD_whole_tree, chao1, observed_otus and Shannon’s Index) and beta diversity (unweighted UniFrac, weighted UniFrac and bray_curtis distance) differences between groups were performed using a two-sided Student’s two-sample t-test embedded in the QIIME 1.9.1 work flow: *core_diversity_analyses.py*. Taxa relative abundance differences over groups of gynecological diagnosis, childbearing history and menstrual cycle were estimated based on parametric ANOVA and nonparametric kruskal_wallis in QIIME 1.9.1 script: *group_significance.py*. The relative abundances of 3 bacterial genera that have significant differences were intensively compared between every 2 groups by Post Hoc Bonferroni multiple comparisons in SPSS (version 22.0). Bonferroni correction was used to adjust for tests of multiple taxa. *P* values less than 0.05 were considered significant after adjustment for multiple tests.

## Supplementary Information

**Additional file 1: Supplementary Material: Figure S1.** The 3 grouping schemes of study subjects, the numbers in the parentheses are sample size of each group. **Table S1.** The comparisons of characteristics among groups by childbearing history, menstrual cycle and gynecological infections respectively. **Table S2.** Comparison of alpha diversities on measure of chao1, observed OTUs, PD_whole tree and Shannon’s index by groups of gynecological diagnosis, childbearing history and menstrual cycle respectively. **Table S3.** Comparison of beta diversities on measure of Unweighted UniFrac distance by groups of gynecological diagnosis, childbearing history and menstrual cycle respectively. The tests of significance were performed using a two-sided Student's two-sample t-test. **Table S4.** The relative abundance of 3 genera differed significantly among groups of gynecological diagnosis by ANOVA. **Table S5.** Bonferroni multiple comparisons of the relative abundance of 3 genera among groups of gynecological diagnosis. **Table S6.** The count and quality of sequence reads from each sample on the Illumina MiSeq platform.

## Data Availability

The datasets used and/or analysed during the current study are available from the corresponding author on reasonable request.

## Abbreviations

ANOVA: Analysis of variance
ASVs: Amplicon sequence variants
BV: Bacterial vaginosis
BVAB: Bacterial vaginosis associated bacteria
IQR: Interquartile range
MV: Mycotic vaginitis
NUC: Nongonococcal urethritis/cervicitis
OTU: Operational taxonomical unit
PCoA: Principal coordinates analysis
PD_whole_tree: Phylogenetic diversity whole tree
QIIME: Quantitative insights into microbial ecology
