# Supplementary material for: Reproductive tract microbiota of women in childbearing age shifts upon gynecological infections and menstrual cycle
Source: BMC Microbiol. 2021 Sep 21;21:252. doi: 10.1186/s12866-021-02300-4 (PMC8454066; doi:10.1186/s12866-021-02300-4)
Supplement: Supplementary file 1 — Additional file 1: Supplementary Material: Figure S1. The 3 grouping schemes of study subjects, the numbers in the parentheses are sample size of each group. Table S1. The comparisons of characteristics among groups by childbearing history, menstrual cycle and gynecological infections respectively. Table S2. Comparison of alpha diversities on measure of chao1, observed OTUs, PD_whole tree and Shannon’s index by groups of gynecological diagnosis, childbearing history and menstrual cycle respectively. Table S3. Comparison of beta diversities on measure of Unweighted UniFrac distance by groups of gynecological diagnosis, childbearing history and menstrual cycle respectively. The tests of significance were performed using a two-sided Student's two-sample t-test. Table S4. The relative abundance of 3 genera differed significantly among groups of gynecological diagnosis by ANOVA. Table S5. Bonferroni multiple comparisons of the relative abundance of 3 genera among groups of gynecological diagnosis. Table S6. The count and quality of sequence reads from each sample on the Illumina MiSeq platform. [file 12866_2021_2300_MOESM1_ESM.docx]

Supplementary Material


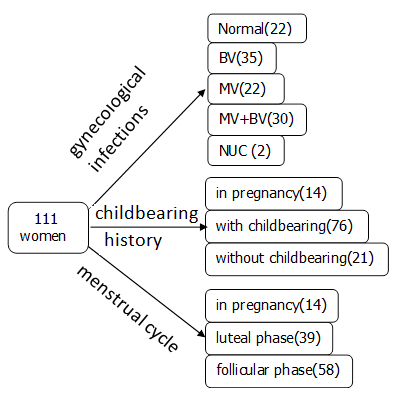


Figure S1: The 3 grouping schemes of study subjects, the numbers in the parentheses are sample size of each group. BV: bacterial vaginosis; MV: mycotic vaginitis; NUC: nongonococcal urethritis/cervicitis

Table S1: the comparisons of characteristics among groups by childbearing history, menstrual cycle and gynecological infections respectively.

| The comparison of characteristics by childbearing history using one-way ANOVA | | | | | |
| --- | --- | --- | --- | --- | --- |
| Characteristic Group | | *n* | Mean | Std. Deviation | *P*_value |
|  |  |  |  |  |  |
| age | With_childbearing | 76 | 35.80 | 6.639 |  |
|  | Without_childbearing | 21 | 22.76 | 1.972 | 0.000 |
|  | In_pregnancy | 14 | 30.07 | 5.030 |  |
|  | Total | 111 | 32.61 | 7.754 |  |
| age_at_first_marriage | With_childbearing | 59 | 24.73 | 2.333 |  |
|  | Without_childbearing | 3 | 23.00 | 3.000 | 0.457 |
|  | In_pregnancy | 12 | 24.75 | 2.221 |  |
|  | Total | 74 | 24.66 | 2.331 |  |
| Days of menstrual_cycle | With_childbearing | 76 | 30.37 | 5.286 |  |
|  | Without_childbearing | 21 | 29.38 | 1.396 | 0.613 |
|  | In_pregnancy | 14 | 30.79 | 3.534 |  |
|  | Total | 111 | 30.23 | 4.590 |  |
| pH | With_childbearing | 76 | 4.288 | .6190 |  |
|  | Without_childbearing | 21 | 4.295 | .4653 | 0.063 |
|  | In_pregnancy | 14 | 3.893 | .5327 |  |
|  | Total | 111 | 4.240 | .5930 |  |
| The comparison of characteristics by menstrual phase using one-way ANOVA | | | | | |
| Characteristic Group | | *n* | Mean | Std. Deviation | *P*_value |
|  |  |  |  |  |  |
| age | Luteal_phase | 39 | 32.18 | 8.309 |  |
|  | Follicular_phase | 58 | 33.52 | 7.854 | 0.302 |
|  | In_pregnancy | 14 | 30.07 | 5.030 |  |
|  | Total | 111 | 32.61 | 7.754 |  |
| age_at_first_marriage | Luteal_phase | 27 | 24.70 | 2.493 |  |
|  | Follicular_phase | 35 | 24.60 | 2.303 | 0.976 |
|  | In_pregnancy | 12 | 24.75 | 2.221 |  |
|  | Total | 74 | 24.66 | 2.331 |  |
| Days of menstrual_cycle | Luteal_phase | 39 | 29.38 | 3.032 |  |
|  | Follicular_phase | 58 | 30.67 | 5.561 | 0.359 |
|  | In_pregnancy | 14 | 30.79 | 3.534 |  |
|  | Total | 111 | 30.23 | 4.590 |  |
| pH | Luteal_phase | 39 | 4.315 | .5869 |  |
|  | Follicular_phase | 58 | 4.272 | .5914 | 0.059 |
|  | In_pregnancy | 14 | 3.893 | .5327 |  |
|  | Total | 111 | 4.240 | .5930 |  |
| The comparison of characteristics by gynecological infections using one-way ANOVA | | | | | |
| Characteristic Group | | *n* | Mean | Std. Deviation | *P*_value |
|  |  |  |  |  |  |
| age | BV | 35 | 34.09 | 7.497 |  |
|  | MV | 22 | 33.32 | 8.747 |  |
|  | MV+BV | 30 | 31.53 | 7.938 | 0.577 |
|  | NUC | 2 | 32.50 | 2.121 |  |
|  | Normal | 22 | 31.05 | 7.201 |  |
|  | Total | 111 | 32.61 | 7.754 |  |
| age_at_first_marriage | BV | 25 | 25.20 | 2.566 |  |
|  | MV | 9 | 24.44 | 1.333 |  |
|  | MV+BV | 21 | 24.29 | 2.849 | 0.655 |
|  | NUC | 1 | 23.00 | . |  |
|  | Normal | 18 | 24.56 | 1.688 |  |
|  | Total | 74 | 24.66 | 2.331 |  |
| Days of menstrual_cycle | BV | 35 | 30.86 | 4.870 |  |
|  | MV | 22 | 28.95 | 1.731 |  |
|  | MV+BV | 30 | 29.23 | 2.674 | 0.124* |
|  | NUC | 2 | 42.50 | 17.678 |  |
|  | Normal | 22 | 30.77 | 5.080 |  |
|  | Total | 111 | 30.23 | 4.590 |  |
| pH | BV | 35 | 4.229 | .5470 |  |
|  | MV | 22 | 4.255 | .7842 |  |
|  | MV+BV | 30 | 4.397 | .3605 | 0.081 |
|  | NUC | 2 | 4.800 | .2828 |  |
|  | Normal | 22 | 3.977 | .6495 |  |
|  | Total | 111 | 4.240 | .5930 |  |

*Tested by nonparametric Kruskal-Wallis because data did not meet the prerequisite for ANOVA.

Normal=without any gynecological infections; BV=bacterial vaginosis; MV=mmycotic vaginitis; MV+BV= simultaneously affected by MV and BV; NUC= nongonococcal urethritis/cervicitis

Table S2: Comparison of alpha diversities on measure of chao1, observed OTUs, PD_whole tree and Shannon’s index by groups of gynecological diagnosis, childbearing history and menstrual cycle respectively.

| **Compare alpha diversity on measure of chao1** | | | | | | | |
| --- | --- | --- | --- | --- | --- | --- | --- |
| Grouped by gynecological diagnosis | | | | | | | |
| Group1 | Group2 | Group1 mean | Group1 std | Group2 mean | Group2 std | *t* stat | *P*-value |
| NUC | Normal | 28 | 17 | 20.84545 | 8.787218 | 0.952265 | 1 |
| MV | NUC | 25.59114 | 10.14995 | 28 | 17 | -0.28684 | 1 |
| MV | Normal | 25.59114 | 10.14995 | 20.84545 | 8.787218 | 1.619894 | 1 |
| BV | Normal | 28.41071 | 14.25675 | 20.84545 | 8.787218 | 2.196644 | 0.35 |
| BV | NUC | 28.41071 | 14.25675 | 28 | 17 | 0.038107 | 1 |
| MV+BV | Normal | 26.29011 | 12.27919 | 20.84545 | 8.787218 | 1.738832 | 0.86 |
| BV | MV | 28.41071 | 14.25675 | 25.59114 | 10.14995 | 0.793529 | 1 |
| MV+BV | NUC | 26.29011 | 12.27919 | 28 | 17 | -0.17955 | 1 |
| MV+BV | MV | 26.29011 | 12.27919 | 25.59114 | 10.14995 | 0.213692 | 1 |
| MV+BV | BV | 26.29011 | 12.27919 | 28.41071 | 14.25675 | -0.62711 | 1 |
| Grouped by childbearing history | | | | | | | |

| Group1 | Group2 | Group1 mean | Group1 std | Group2 mean | Group2 std | t stat | p-value |
| --- | --- | --- | --- | --- | --- | --- | --- |
| in_pregnancy | with_childbearing | 20.3 | 12.35655 | 26.29419 | 11.74688 | -1.72072 | 0.246 |
| in_pregnancy | without_childbearing | 20.3 | 12.35655 | 27.52976 | 13.53527 | -1.55594 | 0.396 |
| with_childbearing | without_childbearing | 26.29419 | 11.74688 | 27.52976 | 13.53527 | -0.40801 | 1 |

| Grouped by menstrual cycle |
| --- |

| Group1 | Group2 | Group1 mean | Group1 std | Group2 mean | Group2 std | t stat | p-value |
| --- | --- | --- | --- | --- | --- | --- | --- |
| Luteal_phase | in_pregnancy | 25.83226 | 11.25758 | 20.3 | 12.35655 | 1.507039 | 0.432 |
| Luteal_phase | follicular_phase | 25.83226 | 11.25758 | 27.05216 | 12.71863 | -0.47973 | 1 |
| follicular_phase | in_pregnancy | 27.05216 | 12.71863 | 20.3 | 12.35655 | 1.767582 | 0.249 |

| **Compare alpha diversity on measure of observed_outs** | | | | | | | |
| --- | --- | --- | --- | --- | --- | --- | --- |
| Grouped by gynecological diagnosis | | | | | | | |
| Group1 | Group2 | Group1 mean | Group1 std | Group2 mean | Group2 std | t stat | p-value |
| NUC | Normal | 28 | 17 | 20.84545 | 8.787218 | 0.952265 | 1 |
| MV | NUC | 25.51364 | 9.999832 | 28 | 17 | -0.2996 | 1 |
| MV | Normal | 25.51364 | 9.999832 | 20.84545 | 8.787218 | 1.606982 | 1 |
| BV | Normal | 28.34 | 14.23995 | 20.84545 | 8.787218 | 2.178184 | 0.3 |
| BV | NUC | 28.34 | 14.23995 | 28 | 17 | 0.03158 | 1 |
| MV+BV | Normal | 26.28 | 12.27614 | 20.84545 | 8.787218 | 1.735916 | 1 |
| BV | MV | 28.34 | 14.23995 | 25.51364 | 9.999832 | 0.798995 | 1 |
| MV+BV | NUC | 26.28 | 12.27614 | 28 | 17 | -0.18065 | 1 |
| MV+BV | MV | 26.28 | 12.27614 | 25.51364 | 9.999832 | 0.23549 | 1 |
| MV+BV | BV | 26.28 | 12.27614 | 28.34 | 14.23995 | -0.60968 | 1 |
| NUC | Normal | 28 | 17 | 20.84545 | 8.787218 | 0.952265 | 1 |
| Grouped by childbearing history | | | | | | | |

| Group1 | Group2 | Group1 mean | Group1 std | Group2 mean | Group2 std | t stat | p-value |
| --- | --- | --- | --- | --- | --- | --- | --- |
| in_pregnancy | with_childbearing | 20.3 | 12.35655 | 26.25263 | 11.70359 | -1.71403 | 0.291 |
| in_pregnancy | without_childbearing | 20.3 | 12.35655 | 27.46667 | 13.51219 | -1.54406 | 0.363 |
| with_childbearing | without_childbearing | 26.25263 | 11.70359 | 27.46667 | 13.51219 | -0.40217 | 1 |

| Grouped by menstrual cycle |
| --- |

| Group1 | Group2 | Group1 mean | Group1 std | Group2 mean | Group2 std | t stat | p-value |
| --- | --- | --- | --- | --- | --- | --- | --- |
| Luteal_phase | in_pregnancy | 25.77949 | 11.18213 | 20.3 | 12.35655 | 1.499671 | 0.438 |
| Luteal_phase | follicular_phase | 25.77949 | 11.18213 | 27.01034 | 12.70118 | -0.4856 | 1 |
| follicular_phase | in_pregnancy | 27.01034 | 12.70118 | 20.3 | 12.35655 | 1.758602 | 0.264 |

| **Compare alpha diversity on measure of PD_whole tree** | | | | | | | |
| --- | --- | --- | --- | --- | --- | --- | --- |
| Grouped by gynecological diagnosis | | | | | | | |
| Group1 | Group2 | Group1 mean | Group1 std | Group2 mean | Group2 std | t stat | p-value |
| NUC | Normal | 3.162241 | 2.066072 | 2.241313 | 1.059462 | 1.014572 | 1 |
| MV | NUC | 2.931085 | 1.368294 | 3.162241 | 2.066072 | -0.20818 | 1 |
| MV | Normal | 2.931085 | 1.368294 | 2.241313 | 1.059462 | 1.826583 | 0.81 |
| BV | Normal | 3.48518 | 1.551421 | 2.241313 | 1.059462 | 3.248462 | 0.04 |
| BV | NUC | 3.48518 | 1.551421 | 3.162241 | 2.066072 | 0.272821 | 1 |
| MV+BV | Normal | 3.484177 | 1.889122 | 2.241313 | 1.059462 | 2.727665 | 0.07 |
| BV | MV | 3.48518 | 1.551421 | 2.931085 | 1.368294 | 1.348563 | 1 |
| MV+BV | NUC | 3.484177 | 1.889122 | 3.162241 | 2.066072 | 0.224569 | 1 |
| MV+BV | MV | 3.484177 | 1.889122 | 2.931085 | 1.368294 | 1.14433 | 1 |
| MV+BV | BV | 3.484177 | 1.889122 | 3.48518 | 1.551421 | -0.00231 | 1 |
| Grouped by childbearing history | | | | | | | |

| Group1 | Group2 | Group1 mean | Group1 std | Group2 mean | Group2 std | t stat | p-value |
| --- | --- | --- | --- | --- | --- | --- | --- |
| in_pregnancy | with_childbearing | 2.095598 | 0.82963 | 3.293434 | 1.587908 | -2.72335 | 0.021 |
| in_pregnancy | without_childbearing | 2.095598 | 0.82963 | 3.189737 | 1.87465 | -1.9943 | 0.159 |
| with_childbearing | without_childbearing | 3.293434 | 1.587908 | 3.189737 | 1.87465 | 0.251642 | 1 |

| Grouped by menstrual cycle |
| --- |

| Group1 | Group2 | Group1 mean | Group1 std | Group2 mean | Group2 std | t stat | p-value |
| --- | --- | --- | --- | --- | --- | --- | --- |
| Luteal_phase | in_pregnancy | 3.343018 | 1.746058 | 2.095598 | 0.82963 | 2.521993 | 0.075 |
| Luteal_phase | follicular_phase | 3.343018 | 1.746058 | 3.222548 | 1.588587 | 0.348143 | 1 |
| follicular_phase | in_pregnancy | 3.222548 | 1.588587 | 2.095598 | 0.82963 | 2.535105 | 0.048 |

| **Compare alpha diversity on measure of Shanonn’s index** | | | | | | | |
| --- | --- | --- | --- | --- | --- | --- | --- |
| Grouped by gynecological diagnosis | | | | | | | |
| Group1 | Group2 | Group1 mean | Group1 std | Group2 mean | Group2 std | t stat | p-value |
| NUC | Normal | 2.412418 | 0.684559 | 2.325036 | 0.623305 | 0.180197 | 1 |
| MV | NUC | 2.478004 | 0.642012 | 2.412418 | 0.684559 | 0.131684 | 1 |
| MV | Normal | 2.478004 | 0.642012 | 2.325036 | 0.623305 | 0.783393 | 1 |
| BV | Normal | 2.599098 | 0.959166 | 2.325036 | 0.623305 | 1.170282 | 1 |
| BV | NUC | 2.599098 | 0.959166 | 2.412418 | 0.684559 | 0.26389 | 1 |
| MV+BV | Normal | 2.514869 | 0.6271 | 2.325036 | 0.623305 | 1.060235 | 1 |
| BV | MV | 2.599098 | 0.959166 | 2.478004 | 0.642012 | 0.513814 | 1 |
| MV+BV | NUC | 2.514869 | 0.6271 | 2.412418 | 0.684559 | 0.21532 | 1 |
| MV+BV | MV | 2.514869 | 0.6271 | 2.478004 | 0.642012 | 0.20331 | 1 |
| MV+BV | BV | 2.514869 | 0.6271 | 2.599098 | 0.959166 | -0.40509 | 1 |
| Grouped by childbearing history | | | | | | | |

| Group1 | Group2 | Group1 mean | Group1 std | Group2 mean | Group2 std | t stat | p-value |
| --- | --- | --- | --- | --- | --- | --- | --- |
| in_pregnancy | with_childbearing | 2.291873 | 0.911447 | 2.503097 | 0.725153 | -0.94849 | 1 |
| in_pregnancy | without_childbearing | 2.291873 | 0.911447 | 2.599269 | 0.730003 | -1.07133 | 0.858 |
| with_childbearing | without_childbearing | 2.503097 | 0.725153 | 2.599269 | 0.730003 | -0.53161 | 1 |

| Grouped by menstrual cycle |
| --- |

| Group1 | Group2 | Group1 mean | Group1 std | Group2 mean | Group2 std | t stat | p-value |
| --- | --- | --- | --- | --- | --- | --- | --- |
| Luteal_phase | in_pregnancy | 2.447076 | 0.55122 | 2.291873 | 0.911447 | 0.734165 | 1 |
| Luteal_phase | follicular_phase | 2.447076 | 0.55122 | 2.575587 | 0.820771 | -0.84763 | 1 |
| follicular_phase | in_pregnancy | 2.575587 | 0.820771 | 2.291873 | 0.911447 | 1.119501 | 0.807 |

| Group 1 | Group 2 | t statistic | Parametric p-value | Parametric p-value (Bonferroni-corrected) | Nonparametric p-value | Nonparametric p-value (Bonferroni-corrected) |
| --- | --- | --- | --- | --- | --- | --- |
| Within Normal | Normal vs. BV | -6.32767 | 3.75E-10 | 5.10E-08 | 0.001 | 0.136 |
| Within Normal | Normal vs. MV | -4.44422 | 1.02E-05 | 0.001391 | 0.001 | 0.136 |
| Within Normal | Normal vs. MV+BV | -6.87124 | 1.20E-11 | 1.63E-09 | 0.001 | 0.136 |
| Within Normal | Normal vs. NUC | -2.53734 | 0.011727 | 1 | 0.011 | 1 |
| Within in_pregnancy | Luteal_phase vs. in_pregnancy | -6.5738 | 8.97E-11 | 2.51E-09 | 0.001 | 0.028 |
| Within in_pregnancy | follicular_phase vs. in_pregnancy | -6.47012 | 1.48E-10 | 4.14E-09 | 0.001 | 0.028 |
| Within in_pregnancy | in_pregnancy vs. with_childbearing | -4.70404 | 2.86E-06 | 8.01E-05 | 0.001 | 0.028 |
| Within in_pregnancy | in_pregnancy vs. without_childbearing | -4.20476 | 3.26E-05 | 0.000912 | 0.001 | 0.028 |

Table S3: Comparison of beta diversities on measure of Unweighted UniFrac distance by groups of gynecological diagnosis, childbearing history and menstrual cycle respectively. The tests of significance were performed using a two-sided Student's two-sample t-test.

Table S4: The relative abundance of 3 genera differed significantly among groups of gynecological diagnosis by ANOVA.

| OTU | Test-Statistic | P | FDR_P | Bonferroni_P | Normal_mean | BV_mean | MV_mean | NUC_mean | MV+BV_mean |
| --- | --- | --- | --- | --- | --- | --- | --- | --- | --- |
| k__Bacteria;p__Firmicutes;c__Bacilli;o__Lactobacillales;f__Streptococcaceae;g__Streptococcus | 17.95968 | 2.76E-11 | 3.03E-10 | 3.03E-10 | 0.000996 | 0.011424 | 0.003054 | 0.343615 | 0.00537 |
| k__Bacteria;p__Firmicutes;c__Bacilli;o__Lactobacillales;Other;Other | 3.270676 | 0.014295 | 0.065923 | 0.157246 | 0.000518 | 0.001164 | 0.014894 | 0.000238 | 0.008587 |
| k__Bacteria;p__Actinobacteria;c__Actinobacteria;o__Bifidobacteriales;f__Bifidobacteriaceae;g__Gardnerella | 3.122475 | 0.017979 | 0.065923 | 0.197769 | 0.041916 | 0.091513 | 0.268824 | 0.010226 | 0.207223 |

Table S5: Bonferroni multiple comparisons of the relative abundance of 3 genera among groups of gynecological diagnosis.

| Dependent Variable | (I) group | (J) group | Mean Difference (I-J) | Std. Error | Sig | 95% Confidence Interval | |
| --- | --- | --- | --- | --- | --- | --- | --- |
|  |  |  |  |  |  | Lower Bound | Upper Bound |
| k__Bacteria;p__Actinobacteria;c__Actinobacteria;o__Bifidobacteriales;f__Bifidobacteriaceae;g__Gardnerella | BV | MV | -.177311192065 | .0698 58578333 | .126 | -.37759432230 | .02297193817 |
|  |  | MV+BV | -.115710728195 | .063883647810 | .729 | -.29886385355 | .06744239716 |
|  |  | NUC | .081286623071 | .186672145916 | 1.000 | -.45389864128 | .61647188742 |
|  |  | Normal | .049596802753 | .069858578333 | 1.000 | -.15068632749 | .24987993299 |
|  | MV | BV | .177311192065 | .069858578333 | .126 | -.02297193817 | .37759432230 |
|  |  | MV+BV | .061600463870 | .072070474604 | 1.000 | -.14502412821 | .26822505595 |
|  |  | NUC | .258597815136 | .189629963120 | 1.000 | -.28506745127 | .80226308155 |
|  |  | Normal | .226907994818^*^ | .077416108264 | .041 | .00495757922 | .44885841041 |
|  | MV+BV | BV | .115710728195 | .063883647810 | .729 | -.06744239716 | .29886385355 |
|  |  | MV | -.061600463870 | .072070474604 | 1.000 | -.26822505595 | .14502412821 |
|  |  | NUC | .196997351267 | .187511126080 | 1.000 | -.34059325223 | .73458795477 |
|  |  | Normal | .165307530948 | .072070474604 | .238 | -.04131706113 | .37193212303 |
|  | NUC | BV | -.081286623071 | .186672145916 | 1.000 | -.61647188742 | .45389864128 |
|  |  | MV | -.258597815136 | .189629963120 | 1.000 | -.80226308155 | .28506745127 |
|  |  | MV+BV | -.196997351267 | .187511126080 | 1.000 | -.73458795477 | .34059325223 |
|  |  | Normal | -.031689820318 | .189629963120 | 1.000 | -.57535508673 | .51197544609 |
|  | Normal | BV | -.049596802753 | .069858578333 | 1.000 | -.24987993299 | .15068632749 |
|  |  | MV | -.226907994818^*^ | .077416108264 | .041 | -.44885841041 | -.00495757922 |
|  |  | MV+BV | -.165307530948 | .072070474604 | .238 | -.37193212303 | .04131706113 |
|  |  | NUC | .031689820318 | .189629963120 | 1.000 | -.51197544609 | .57535508673 |
| k__Bacteria;p__Firmicutes;c__Bacilli;o__Lactobacillales;f__Streptococcaceae;g__Streptococcus | BV | MV | .008370339855 | .015250055474 | 1.000 | -.03535126055 | .05209194026 |
|  |  | MV+BV | .006054347300 | .013945734314 | 1.000 | -.03392779073 | .04603648533 |
|  |  | NUC | -.332191407100^*^ | .040750336587 | .000 | -.44902179736 | -.21536101684 |
|  |  | Normal | .010428087582 | .015250055474 | 1.000 | -.03329351282 | .05414968798 |
|  | MV | BV | -.008370339855 | .015250055474 | 1.000 | -.05209194026 | .03535126055 |
|  |  | MV+BV | -.002315992555 | .015732910144 | 1.000 | -.04742192753 | .04278994242 |
|  |  | NUC | -.340561746955^*^ | .041396025027 | .000 | -.45924331291 | -.22188018100 |
|  |  | Normal | .002057747727 | .016899856449 | 1.000 | -.04639379868 | .05050929414 |
|  | MV+BV | BV | -.006054347300 | .013945734314 | 1.000 | -.04603648533 | .03392779073 |
|  |  | MV | .002315992555 | .015732910144 | 1.000 | -.04278994242 | .04742192753 |
|  |  | NUC | -.338245754400^*^ | .040933485090 | .000 | -.45560122772 | -.22089028108 |
|  |  | Normal | .004373740282 | .015732910144 | 1.000 | -.04073219469 | .04947967525 |
|  | NUC | BV | .332191407100^*^ | .040750336587 | .000 | .21536101684 | .44902179736 |
|  |  | MV | .340561746955^*^ | .041396025027 | .000 | .22188018100 | .45924331291 |
|  |  | MV+BV | .338245754400^*^ | .040933485090 | .000 | .22089028108 | .45560122772 |
|  |  | Normal | .342619494682^*^ | .041396025027 | .000 | .22393792873 | .46130106063 |
|  | Normal | BV | -.010428087582 | .015250055474 | 1.000 | -.05414968798 | .03329351282 |
|  |  | MV | -.002057747727 | .016899856449 | 1.000 | -.05050929414 | .04639379868 |
|  |  | MV+BV | -.004373740282 | .015732910144 | 1.000 | -.04947967525 | .04073219469 |
|  |  | NUC | -.342619494682^*^ | .041396025027 | .000 | -.46130106063 | -.22393792873 |
| k__Bacteria;p__Firmicutes;c__Bacilli;o__Lactobacillales;Other;Other | BV | MV | -.013730734082^*^ | .004437624064 | .025 | -.02645331246 | -.00100815570 |
|  |  | MV+BV | -.007423123500 | .004058078759 | .702 | -.01905755317 | .00421130617 |
|  |  | NUC | .000925893100 | .011857968293 | 1.000 | -.03307066201 | .03492244821 |
|  |  | Normal | .000645755600 | .004437624064 | 1.000 | -.01207682278 | .01336833398 |
|  | MV | BV | .013730734082^*^ | .004437624064 | .025 | .00100815570 | .02645331246 |
|  |  | MV+BV | .006307610582 | .004578130275 | 1.000 | -.00681779626 | .01943301743 |
|  |  | NUC | .014656627182 | .012045857614 | 1.000 | -.01987860280 | .04919185717 |
|  |  | Normal | .014376489682^*^ | .004917700778 | .042 | .00027754108 | .02847543828 |
|  | MV+BV | BV | .007423123500 | .004058078759 | .702 | -.00421130617 | .01905755317 |
|  |  | MV | -.006307610582 | .004578130275 | 1.000 | -.01943301743 | .00681779626 |
|  |  | NUC | .008349016600 | .011911262801 | 1.000 | -.02580033278 | .04249836598 |
|  |  | Normal | .008068879100 | .004578130275 | .809 | -.00505652774 | .02119428594 |
|  | NUC | BV | -.000925893100 | .011857968293 | 1.000 | -.03492244821 | .03307066201 |
|  |  | MV | -.014656627182 | .012045857614 | 1.000 | -.04919185717 | .01987860280 |
|  |  | MV+BV | -.008349016600 | .011911262801 | 1.000 | -.04249836598 | .02580033278 |
|  |  | Normal | -.000280137500 | .012045857614 | 1.000 | -.03481536749 | .03425509249 |
|  | Normal | BV | -.000645755600 | .004437624064 | 1.000 | -.01336833398 | .01207682278 |
|  |  | MV | -.014376489682^*^ | .004917700778 | .042 | -.02847543828 | -.00027754108 |
|  |  | MV+BV | -.008068879100 | .004578130275 | .809 | -.02119428594 | .00505652774 |
|  |  | NUC | .000280137500 | .012045857614 | 1.000 | -.03425509249 | .03481536749 |
| *. The mean difference is significant at the 0.05 level. | | | | | | | |

Table S6: The count and quality of sequence reads from each sample on the Illumina MiSeq platform.

| Lane | SampleID | Index | Reads | Yield(Gbases) | Q30(%) |
| --- | --- | --- | --- | --- | --- |
| 1 | a161 | CGGAGCCT+TGGATATC | 74,843 | 0.03742 | 88.47 |
| 1 | a68 | GCTCATGA+CTAAGCCT | 65,449 | 0.03272 | 82.07 |
| 1 | a109 | GCTGGAAT+TGCCGACA | 46,928 | 0.02346 | 90.21 |
| 1 | a20 | TACGCTGC+GACAACCA | 57,440 | 0.02872 | 89.14 |
| 1 | a71 | CTCCGATT+CTAAGCCT | 64,713 | 0.03236 | 88.71 |
| 1 | a63 | AGTTGTCC+GACAACCA | 51,100 | 0.02555 | 94.18 |
| 1 | a76 | CTCCGATT+AGTCACGA | 126,809 | 0.0634 | 89.01 |
| 1 | a41 | GACTGGAG+AGGTGTTG | 68,807 | 0.0344 | 89.19 |
| 1 | a42 | GACTGGAG+GACTGGTC | 48,713 | 0.02436 | 83.86 |
| 1 | a153 | TTCAAGCT+TACGAAGC | 44,224 | 0.02211 | 82.81 |
| 1 | a160 | CGGAGCCT+CAAGCCTC | 101,769 | 0.05088 | 88.96 |
| 1 | a155 | TTCAAGCT+CAGCACAG | 40,406 | 0.0202 | 85.43 |
| 1 | a13 | TAGGCATG+AGTCACGA | 50,511 | 0.02526 | 84.68 |
| 1 | a170 | ACTGAGCG+TGGATATC | 63,170 | 0.03159 | 88.64 |
| 1 | a80 | CTCCGATT+TCAGTTGT | 60,340 | 0.03017 | 92.3 |
| 1 | a168 | ACTGAGCG+CTGCTTCC | 51,824 | 0.02591 | 89.44 |
| 1 | a60 | TAACCGAT+TCAGTTGT | 65,888 | 0.03294 | 88.56 |
| 1 | a51 | TAACCGAT+AAGGCTAT | 174,385 | 0.08719 | 87.36 |
| 1 | a74 | CTCCGATT+GACAACCA | 63,345 | 0.03167 | 89.88 |
| 1 | a133 | TTCAAGCT+CCTAGAGT | 48,312 | 0.02416 | 90.62 |
| 1 | a18 | TACGCTGC+CTAAGCCT | 70,492 | 0.03525 | 86.67 |
| 1 | a64 | AGTTGTCC+AGTCACGA | 121,869 | 0.06093 | 87.84 |
| 1 | a85 | ACAATCGG+AGTCACGA | 57,651 | 0.02883 | 91.58 |
| 1 | a105 | GCTCATGA+AGGTGTTG | 66,966 | 0.03348 | 80.96 |
| 1 | a7 | AGGCAGAA+GACTGGTC | 166,156 | 0.08308 | 71.78 |
| 1 | a34 | GACTGGAG+AAGGCTAT | 42,079 | 0.02104 | 86.38 |
| 1 | a6 | GCAACACA+TCGACTAG | 81,153 | 0.04058 | 82.8 |
| 1 | a44 | GACTGGAG+CCTGAGAC | 57,715 | 0.02886 | 90.88 |
| 1 | a162 | CAGCTTGG+TGCCGACA | 50,917 | 0.02546 | 79.99 |
| 1 | a164 | CGGAGCCT+ACCTTCAA | 73,899 | 0.03695 | 91.15 |
| 1 | a169 | ACTGAGCG+CAAGCCTC | 64,613 | 0.03231 | 85.8 |
| 1 | a10 | GTAGAGGA+AGTCACGA | 66,881 | 0.03344 | 80.39 |
| 1 | a72 | CTCCGATT+AAGGCTAT | 61,032 | 0.03052 | 87.85 |
| 1 | a111 | GCTCATGA+TCAGTTGT | 69,550 | 0.03478 | 79.9 |
| 1 | a100 | CTGGTGAT+GACTGGTC | 51,459 | 0.02573 | 83.93 |
| 1 | a156 | CAGCTTGG+CCTAGAGT | 44,698 | 0.02235 | 80.42 |
| 1 | a114 | GCTGGAAT+AGTACGCG | 45,283 | 0.02264 | 91.73 |
| 1 | a135 | TTCAAGCT+GAGCCTTA | 40,173 | 0.02009 | 90.94 |
| 1 | a81 | ACAATCGG+CTAAGCCT | 58,311 | 0.02916 | 87.14 |
| 1 | a78 | CTCCGATT+GACTGGTC | 63,798 | 0.0319 | 90.56 |
| 1 | a57 | TAACCGAT+GACTGGTC | 59,255 | 0.02963 | 86.41 |
| 1 | a50 | TAACCGAT+CTAAGCCT | 71,447 | 0.03572 | 92.02 |
| 1 | a19 | TACGCTGC+AAGGCTAT | 54,763 | 0.02738 | 87.48 |
| 1 | a152 | CGGAGCCT+GTAAGGAG | 68,698 | 0.03435 | 93.21 |
| 1 | a49 | GACTGGAG+TCAGTTGT | 76,743 | 0.03837 | 89.27 |
| 1 | a145 | TGGTACGG+AGTCACGA | 47,613 | 0.02381 | 83.92 |
| 1 | a17 | GTAGAGGA+CCTGAGAC | 62,533 | 0.03127 | 89.64 |
| 1 | a62 | AGTTGTCC+AAGGCTAT | 64,562 | 0.03228 | 89.52 |
| 1 | a70 | AGTTGTCC+TCAGTTGT | 68,210 | 0.03411 | 84.34 |
| 1 | a91 | ACAATCGG+CCTGAGAC | 53,369 | 0.02668 | 88.9 |
| 1 | a167 | ACTGAGCG+ACTGCATA | 62,426 | 0.03121 | 89.66 |
| 1 | a165 | CGGAGCCT+CGAGGTAA | 71,131 | 0.03557 | 91.65 |
| 1 | a61 | AGTTGTCC+CTAAGCCT | 65,525 | 0.03276 | 92.06 |
| 1 | a39 | GACTGGAG+AGTCACGA | 55,741 | 0.02787 | 89.78 |
| 1 | a88 | ACAATCGG+AGGTGTTG | 62,216 | 0.03111 | 87.31 |
| 1 | a5 | AGGCAGAA+AGTCACGA | 59,434 | 0.02972 | 72.71 |
| 1 | a67 | AGTTGTCC+GACTGGTC | 56,080 | 0.02804 | 87.67 |
| 1 | a142 | TGGTACGG+AAGGCTAT | 43,589 | 0.02179 | 85.38 |
| 1 | a132 | TAGCGCTC+GACAACCA | 76,434 | 0.03822 | 90.63 |
| 1 | a166 | ACTGAGCG+GTAAGGAG | 48,535 | 0.02427 | 88.75 |
| 1 | a14 | TAGGCATG+AGGTGTTG | 49,106 | 0.02455 | 90.54 |
| 1 | a79 | CTCCGATT+CCTGAGAC | 64,169 | 0.03208 | 93.61 |
| 1 | a26 | TACGCTGC+CCTGAGAC | 68,161 | 0.03408 | 87.01 |
| 1 | a90 | ACAATCGG+GACTGGTC | 53,953 | 0.02698 | 89.52 |
| 1 | a56 | TAACCGAT+AGGTGTTG | 69,238 | 0.03462 | 87.29 |
| 1 | a159 | TAGCGCTC+AGGTGTTG | 56,754 | 0.02838 | 92.14 |
| 1 | a104 | GCTGGAAT+GAGCCTTA | 43,990 | 0.022 | 83.48 |
| 1 | a33 | GACTGGAG+CTAAGCCT | 62,899 | 0.03145 | 86.76 |
| 1 | a157 | CAGCTTGG+GAGCCTTA | 59,560 | 0.02978 | 89.78 |
| 1 | a136 | CGCTTGCA+TCAGTTGT | 54,725 | 0.02736 | 76.48 |
| 1 | a102 | GCTCATGA+GACAACCA | 60,480 | 0.03024 | 80.9 |
| 1 | a23 | TACGCTGC+AGGTGTTG | 62,180 | 0.03109 | 89.57 |
| 1 | a106 | GCTCATGA+GACTGGTC | 51,572 | 0.02579 | 83.17 |
| 1 | a54 | TAACCGAT+GACAACCA | 72,712 | 0.03636 | 88.54 |
| 1 | a86 | GCTGGAAT+CCTAGAGT | 62,360 | 0.03118 | 91.33 |
| 1 | a149 | TTCAAGCT+ATTGGTGC | 49,716 | 0.02486 | 92.33 |
| 1 | a58 | TAACCGAT+CCTGAGAC | 42,265 | 0.02113 | 91.63 |
| 1 | a144 | TGGTACGG+GACAACCA | 46,250 | 0.02312 | 87.89 |
| 1 | a2 | GTAGAGGA+CTAAGCCT | 58,512 | 0.02926 | 89.44 |
| 1 | a12 | GTAGAGGA+GACTGGTC | 46,909 | 0.02345 | 83.03 |
| 1 | a77 | CTCCGATT+AGGTGTTG | 64,903 | 0.03245 | 87.65 |
| 1 | a128 | TAGCGCTC+AAGGCTAT | 53,484 | 0.02674 | 88.26 |
| 1 | a9 | AGGCAGAA+TCAGTTGT | 173,126 | 0.08656 | 77.82 |
| 1 | a158 | CAGCTTGG+GCATCTCC | 43,659 | 0.02183 | 84.17 |
| 1 | a163 | CGGAGCCT+GAACAGGT | 78,336 | 0.03917 | 89.27 |
| 1 | a36 | GACTGGAG+GACAACCA | 58,721 | 0.02936 | 89.84 |
| 1 | a16 | TAGGCATG+CCTGAGAC | 51,543 | 0.02577 | 89.33 |
| 1 | a8 | AGGCAGAA+CCTGAGAC | 66,887 | 0.03344 | 77.83 |
| 1 | a110 | GCTGGAAT+ATTGGTGC | 50,965 | 0.02548 | 90.92 |
| 1 | a101 | CTGGTGAT+CCTGAGAC | 56,020 | 0.02801 | 79.41 |
| 1 | a99 | GCTCATGA+AAGGCTAT | 54,450 | 0.02722 | 81.1 |
| 1 | a108 | GCTGGAAT+GCATCTCC | 55,571 | 0.02779 | 81.09 |
| 1 | a29 | GTAGAGGA+TCAGTTGT | 65,728 | 0.03286 | 78.76 |
| 1 | a21 | TACGCTGC+AGTCACGA | 103,897 | 0.05195 | 85.41 |
| 1 | a82 | ACAATCGG+AAGGCTAT | 57,537 | 0.02877 | 90.09 |
| 1 | a151 | TGGTACGG+CCTGAGAC | 54,504 | 0.02725 | 87.55 |
| 1 | a125 | CGCTTGCA+AGGTGTTG | 42,988 | 0.02149 | 83.68 |
| 1 | a139 | TAGCGCTC+AGTCACGA | 61,665 | 0.03083 | 92.03 |
| 1 | a154 | CGGAGCCT+ACTGCATA | 67,723 | 0.03386 | 89.03 |
| 1 | a11 | GTAGAGGA+AGGTGTTG | 57,658 | 0.02883 | 86.38 |
| 1 | a177 | ACTGAGCG+CGAGGTAA | 65,422 | 0.03271 | 87.89 |
| 1 | a24 | TACGCTGC+GACTGGTC | 81,431 | 0.04072 | 86.89 |
| 1 | a143 | TTCAAGCT+GCATCTCC | 40,333 | 0.02017 | 81.91 |
| 1 | a171 | ACTGAGCG+GAACAGGT | 61,847 | 0.03092 | 86.69 |
| 1 | a113 | GTTAGCGC+GACTGGTC | 43,466 | 0.02173 | 90.65 |
| 1 | a65 | AGTTGTCC+AGGTGTTG | 45,231 | 0.02262 | 88.93 |
| 1 | a176 | ACTGAGCG+ACCTTCAA | 57,637 | 0.02882 | 87.84 |
| 1 | a178 | GACATATC+GTAAGGAG | 67,427 | 0.03371 | 95.03 |
| 1 | a150 | TTCAAGCT+AGTACGCG | 68,153 | 0.03408 | 92.47 |
| 1 | a83 | ACAATCGG+GACAACCA | 59,976 | 0.02999 | 88.83 |
| 1 | a15 | TAGGCATG+GACTGGTC | 43,379 | 0.02169 | 91.31 |
